# Impact of Soil Field Water Capacity on Secondary Metabolites, Phenylalanine Ammonia-lyase (PAL), Maliondialdehyde (MDA) and Photosynthetic Responses of Malaysian Kacip Fatimah (*Labisia pumila* Benth)

**DOI:** 10.3390/molecules17067305

**Published:** 2012-06-13

**Authors:** Hawa Z. E. Jaafar, Mohd Hafiz Ibrahim, Nur Farhana Mohamad Fakri

**Affiliations:** Department of Crop Science, Faculty of Agriculture, University Putra Malaysia, Serdang 43400, Selangor, Malaysia

**Keywords:** photosynthetic capacity, evapotranspiration replacements, total phenolics and flavonoids, anthocyanins, soluble sugar, lipid peroxidation activity

## Abstract

A randomized complete block design 2 × 4 experiment was designed and conducted for 15 weeks to characterize the relationships between production of total phenolics, flavonoid, anthocyanin, leaf gas exchange, total chlorophyll, phenylalanine ammonia-lyase (PAL) and malondialdehyde (MDA) activity in two varieties of *Labisia pumila* Benth, namely the var. *alata* and *pumila*, under four levels of evapotranspiration replacement (ER) (100%; well watered), (75%, moderate water stress), (50%; high water stress) and (25%; severe water stress). The production of total phenolics, flavonoids, anthocyanin, soluble sugar and relative leaf water content was affected by the interaction between varieties and SWC. As the ER levels decreased from 100% to 25%, the production of PAL and MDA activity increased steadily. At the highest (100%) ER *L. pumila* exhibited signiﬁcantly higher net photosynthesis, apparent quantum yield, maximum efficiency of photosystem II (f_v_/f_m_) and lower dark respiration rates compared to the other treatment. The production of total phenolics, flavonoids and anthocyanin was also found to be higher under high water stress (50% ER replacement) compared to severe water stress (25% ER). From this study, it was observed that as net photosynthesis, apparent quantum yield and chlorophyll content were downregulated under high water stress the production of total phenolics, flavonoids and anthocyanin were upregulated implying that the imposition of high water stress can enhance the medicinal properties of *L. pumila* Benth.

## 1. Introduction

Recently, there has been an increased interest in understanding the mechanism of plant acclimation to environmental stresses [1,2,3]. Plants respond to an adverse ecosystem by altering their morphology, physiology, and biochemistry [2]. Some of the adaptations to stress may include the changes in both the nature and levels of primary and secondary metabolites [4,5]. Recent advances in plant sciences have led to great interest in increasing the production of plant secondary metabolites for their medicinal and aromatic uses [6]. A better understanding of the environmental influences on the regulation of plant secondary metabolism is advantageous for the cultivation of medicinal plants such as *Labisia pumila* Benth [7].

*Labisia pumila* Benth., popularly known as kacip fatimah, is a sub-herbaceous plant with creeping stems from the family Myrsinaceae that is found widespread in Indochina and throughout the Malaysian forest [8]. Traditionally *L. pumila* has been used by Malay women to induce and facilitate childbirth as well as a post-partum medicine. The other uses of this herb are in treatments for dysentery, dysmenorrhea, flatulence, and gonorrhea [9]. This herb has received a lot of attention among scientists, herbalists and the pharmacy industry in Malaysia due to its therapeutic effects and high total contents of phenolic and flavonoid compounds. These polyphenolic compounds have received considerable interest because of their protective role against cancer and heart disease, attributed to their antioxidative activity against reactive oxygen species, which was reported to be higher than that of vitamins C and E [10].

Numerous studies have shown that environmental stress can enhance the production of several secondary metabolites in plants [11,12]. Hence, the production of high levels of secondary metabolites can be induced in plants by manipulating certain specific environmental stress conditions [13]. For instance, the accumulation of plant secondary metabolites can be induced with exposure to nutrient deficiency [14], UV [15], light intensity [16], CO_2_ [17] and temperature [18].

Water stress is one of the most important environmental stresses that can depress growth and alter the biochemical properties of plants [19]. According to Franz [20], Palevitch [21] and Marchese and Figueira [22] one of the most important factors affecting secondary metabolism is soil water capacity. Usually, limited availability of water has a negative effect on plant growth and development. However, an non-severe water deficit has sometimes proven beneficial for the accumulation of biologically-active compounds in medicinal and aromatic plants [21]. Under high water stress, there is a limit on the translocation of carbon to its sinks, with the remaining carbon accumulates as carbohydrates, which leads to an increase in the carbon pool that would be allocated for secondary metabolism, with little or no competition with growth and development [23]. Ghershenzon [4] demonstrated that, in herbaceous plants and shrubs, terpenes tend to increase under stress, mainly under severe water deficit conditions. This type of stress is known to increase the amount of secondary metabolites in a variety of medicinal plants, e.g., artemisinin in *Artemisia annua* L. [24], ajmalicine in *Catharanthus roseus* [25] and hyperforin in *Hypericum perforatum* [19].

There are numerous reports on the biochemical, primary, physiological and morphological responses of *L. pumila* to environmental stress [26,27,28], however fewer studies have been done on microenvironmental manipulation, especially the effects of soil water capacity on the enhancement of secondary metabolite production in *L. pumila*. The effects of soil water capacity on *L. pumila* is an important aspect for the establishment of this plant in field cultivation as recently, this herb has mostly been harvested directly from the forest. Domestication of this herb in greenhouses might provide a consistent supply of raw material and protect the plant from potential extinction due to overharvesting [29]. The study of the impact of water availability on the plant secondary metabolites of *L. pumila* might realize this possibility. The objective of the current study was thus to examine the effects of different soil water capacity levels on production of secondary metabolites (total phenolics, flavonoids and anthocyanin), soluble sugar, total chlorophyll content, leaf gas exchange, phenyll alanine lyase, (PAL) and maliondialdehyde (MDA) activity in two varieties of *L. pumila* (var. *alata* and var. *pumila*) under greenhouse conditions and the determination of the relationships between these parameters.

## 2. Results and Discussion

### 2.1. Total Phenolics and Flavonoids

The interaction effect between ER and varieties had a significant (*p* ≤ 0.05) impact on the production of total phenolics and flavonoids in *L. pumila* (Table 1). As the plant received less water (100% to 25% ER) the production of total phenolics and flavonoids was enhanced. 

**Table 1:** Interaction effects between varieties and evapotranspiration replacement on leaf total phenolics, flavonoids and anthocyanin production.

Varieties	Evapotranspiration	Total Phenolics	Total Flavonoids	Anthocyanin
	replacement	(mg gallic acid/g dry weight)	(mg rutin/g dry weight)	(mg petunidin/ g fresh weight)
***Alata***	100%	0.81 ± 0.01 ^d^	0.52 ± 0.01 ^c^	0.72 ± 0.01 ^d^
	75%	1.23 ± 0.12 ^c^	0.67 ± 0.03 ^b^	0.81 ± 0.02 ^c^
	50%	1.75 ± 0.02 ^b^	0.75 ± 0.13 ^a^	0.92 ± 0.01 ^a^
	25%	1.72 ± 0.14 ^b^	0.64 ± 0.12 ^b^	0.90 ± 0.04 ^a^
***Pumila***	100%	0.79 ± 0.13 ^d^	0.57 ± 0.15 ^c^	0.69 ± 0.01 ^d^
	75%	1.27 ± 0.03 ^c^	0.69 ± 0.16 ^b^	0.79 ± 0.03 ^c^
	50%	1.89 ± 0.03 ^a^	0.75 ± 0.11 ^a^	0.97 ± 0.05 ^a^
	25%	1.83 ± 0.07 ^a^	0.71 ± 0.06 ^a^	0.88 ± 0.04 ^b^

All analyses are mean ± standard error of mean (SEM); N = 30 Means not sharing a common letter within a column were significantly different at *p* ≤ 0.05.

The increase in the production of plant secondary metabolites was found to be statistically higher in var. *pumila* than var. *alata*. The enhancement of total phenolics and flavonoids was optimized when *L. pumila* was exposed to high water stress (50% ER) compared to severe water stress (25% ER) and the control. The present result indicated the production of gallic acid and rutin can be increased with imposition of high water stress (50% ER replacement) compared to severe water stress (25% ER replacement) to *L. pumila*, thus increasing the plant’s medicinal properties. The enhancement of plant medicinal properties under water deficit has been observed by Fortier *et al*. [30] and Azhar *et al.* [31] in *Oreochromis niloticus* and *Trachyspermum ammi*, respectively. They reached a similar conclusion as in the present study, whereby they observed the imposition of high water stress had enhanced production of plant secondary metabolites compared to the imposition of severe water stress. Further, Ibrahim *et al.* [32] attributed the increase in total plant flavonoids and phenolics under high water stress to accumulation of soluble carbohydrates in plants as a result of reduced transportation of soluble sugar under water limitation. A positive significant relationship established between soluble carbohydrate and plant secondary metabolites (Total phenolics; R^2^ = 0.976; Total flavonoids; R^2^ = 0.986; *p* ≤ 0.05) justified these findings (Table 2).

The increase in total production of total phenolics and flavonoids under low soil field water capacity in the current study might be due to an increase in activity of phenylalanine ammonia-lyase (PAL) under low soil field water capacity. Oh *et al.* [33] found that the increase in phenolics and flavonoids compounds in lettuce was due to enhancement of PAL activity. From the correlations in Table 2, it was shown that total phenolics (R^2^ = 0.932; *p* ≤ 0.01) and flavonoids (R^2^ = 0.876; *p* ≤ 0.01) had a significant positive correlation with PAL activity, indicating the increase in production of total phenolics and flavonoids under low soil water capacity might be triggered by high PAL activity. Activation of phenylpropanoid pathways may be dependent on both the type and degree of soil water capacity and the genotype of *Labisia pumila*. According to Harrison and Were [34] the increase in production of secondary metabolites under low soil water capacity might be due to enhanced degradation of larger phenolics compounds into smaller ones as water deficit is enhanced, thus increasing the medicinal properties of the plants.

### 2.2. Anthocyanins

Anthocyanins are probably the largest group of phenolic compounds in the human diet, and their strong antioxidant activities suggest their importance in maintaining health. Anthocyanins are also important as antioxidants, which have roles in promoting good health and reducing the risk of chronic disease and also as anti-inflammatory agents. In the present study, anthocyanin content was found to be influenced by the interaction effects between field capacity and varieties (*p* ≤ 0.01; Table 1). The accumulation of anthocyanin exhibited similar patterns with total phenolics and flavonoids. In the leaves, the imposition of water stress at 50% SWC replacement had increased anthocyanin production to a maximum in both var. *pumila* (0.97 mg/g fresh weight) and var. *alata* (0.92 mg/g fresh weight); whilst the lowest values were recorded under non stress conditions (100% ER) at 0.69 mg/g fresh weight and 0.72 mg/g fresh weight, respectively. The same patterns that were observed in total phenolics and flavonoid accumulation suggest the enhancement of secondary metabolites production in *L. pumila* under high water stress (50% ER) conditions. Acute enhancement of anthocyanin content under high water stress condition was also observed by Pallioti *et al.* [35], where they observed the anthocyanin content was statistically 25% higher in grape fruit that were under high water stress compared to the plant under normal irrigation. 

**Table 2:** Correlations among the measured parameters in the experiments.

Parameters	1	2	3	4	5	6	7	8	9	10	11	12
1.Total phenolics	1.000											
2.Total flavonoids	0.987 *	1.000										
3.Anthocyanin	0.876 *	0.888 *	1.000									
4.Photosynthesis	–0.879 *	–0.855 *	–0.871 *	1.000								
5.AQY	–0.876 *	–0.901 *	–0.899 *	0.978 *	1.000							
6.Dark respiration	0.934 *	0.876 *	0.789 *	0.234	0.113	1.000						
7.f_v_/f_m_	–0.874 *	–0.786 *	–0.699 *	0.766 *	0.433	–0.445	1.000					
8.RWC	–0.854 *	–0.921 *	–0.865 *	0.897 *	0.223	0.778 *	0.899 *	1.000				
9.Carbohydrate	0.976 *	0.986 *	0.765 *	–0.876 *	0.677 *	–0.765 *	–0.556 *	–0.876 *	1.000			
10.Chlorophyll	–0.766 *	–0.786 *	–0.865 *	-0.987 *	0.332	0.344	0.776 *	–0.444	–0.978 *	1.000		
11.PAL	0.932 *	0.876 *	0.812 *	–0.781 *	0.021	0.021	0.034	0.002	0.788 *	0.234	1.000	
12.MDA	0.911 *	0.885 *	0.934 *	–0.886 *	0.032	0.043	0.001 *	0.012	0.876 *	–0.002	0.987 *	1.000

* significant at *p* ≤ 0.05 or *p*≤ 0.01; AQY = Apparent quantum yield; f_v_/f_m_ = maximum efficiency of photosystem II; RWC = relative water content; PAL = phenylalanine ammonia lyase activity; MDA = maliondiadehyde.

**Table 3:** Net photosynthesis, Apparent quantum yield, dark respiration and maximum efficiency of photosystem ii (f_v_/f_m_) under different evapotranspiration replacement status.

Evapotranspiration	Net Photosynthesis	Apparent quantum yield	Dark respiration rate (R_d_)	Maximum efficiency of photosystem II
replacement	(A)	(ɸ)	(µmol/m^2^/s)	(f_v_/f_m_)
	(µmol/m^2^/s)	(µmol/m^2^/s)		
100%	10.75 ± 0.01 ^a^	0.08 ± 0.01 ^a^	4.61 ± 0.21 ^d^	0.84 ± 0.01 ^a^
75%	7.21 ± 0.22 ^b^	0.06 ± 0.02 ^b^	12.71 ± 0.23 ^c^	0.81 ± 0.02 ^b^
50%	3.41 ± 0.12 ^c^	0.04 ± 0.07 ^c^	14.75 ± 0.13 ^b^	0.72 ± 0.06 ^c^
25%	1.72 ± 0.13 ^d^	0.01 ± 0.04 ^d^	15.64 ± 0.14 ^a^	0.66 ± 0.04 ^d^

All analyses are mean ± standard error of mean (SEM); *N* = 60 Means not sharing a common letter within a column are significantly different at *p* ≤ 0.05.

Anthocyanins are the naturally occurring phenolic compounds responsible for the color of many flowers, fruits, and berries [36]. It is the most important group of water soluble pigments in plants and they have beneficial health effects as antioxidant and anti-inflammatory agents [37]. Tamura and Yamagami [38] reported that anthocyanins possess some positive therapeutic value, mainly associated with their antioxidant activities. Improvement in anthocyanin content in the current study with increasing water stress (50% ER) condition suggest a possible way to increase the quality of *L. pumila* by water stressing the plant.

### 2.3. Leaf gas Exchange Properties

In this study, the net photosynthetic rate (A), apparent quantum yield (**ɸ**) and dark respiration rate (R_d_) were determined by a portable infrared photosynthesis system LI-6400 (LI-COR, Lincoln, NE, USA) and Portable Plant Efficient Analyzer (Handy PEA; USA) for maximum efficiency of photosystem II (f_v_/f_m_). In general, the photosynthesis rate (A) was found to be higher in 100% ER (10.75 µmol/m^2^/s), followed by 75% ER (7.21 µmol/m^2^/s), 50% ER (3.41 µmol/m^2^/s) and lowest in 25% ER that just recorded 1.21 µmol/m^2^/s (Table 3). Similar pattern of decreasing values with increasing water stress was also observed with apparent quantum yield (**ɸ**). The latter (**ɸ**) was also found to have a significant positive correlation (Table 2) with net photosynthesis (R^2^ = 0.892; *p* ≤ 0.01), indicating the reduction in photosynthesis rate under high water stress might be followed by the reduction in **ɸ** that indicates that high water stress might reduce the efficiency of photosynthesis by reducing the efficiency of light harvesting of *L. pumila* [29]. However, it was found that as evapotranspiration replacement decreased from 100% to 75% > 50% and 25% the dark respiration rate increased significantly. For instance, as the ER reduced to 75% > 50% and 25% the dark respiration (R_d_) rate enhanced by 175%, 220% and 240% respectively compared to plant under non stress (100% ER). From the correlation analysis (Table 2) was demonstrated that net photosynthesis had a significant negative relationship with total phenolics (R^2^ = −0.876; *p* ≤ 0.05), total flavonoids (R^2^ = −0.855; *p* ≤ 0.05) and anthocyanins content (R^2^ = −0.871; *p* ≤ 0.05) suggesting that the up-regulation of the shikimic acid pathway involved in production of secondary metabolites was under down-regulated photosynthesis [28]. The synthesis of basic skeleton for secondary metabolites is dependent on the carbon assimilated during photosynthesis [39]. In the current study, under low water field capacity the net photosynthesis decreased significantly. Under this condition photosynthesis was limited, probably due to low CO_2_ availability as a result of reduced stomatal and mesophyll conductance. These limited CO_2_ assimilation of the leaf tissues may result in increased allocation of photoassimilates to the production of secondary metabolites. The increase in production of secondary metabolites under low photosynthesis might be due to increased shikimic acid pathway activity under stressed conditions, especially under low water field capacity and low photosynthesis. The increase in production of secondary metabolites under low photosynthetic activity was also observed by Moghadam *et al.* [40] in *Centalia asiatica*. Furthermore, a significant positive correlation of dark respiration rate with total phenolics (R^2^ = 0.934; *p* ≤ 0.05), flavonoids (R^2^ = 0.876; *p* ≤ 0.05) and anthocyanins (R^2^ = 0.789; *p* ≤ 0.05) was observed; which indicates induction of high respiration and low photosynthetic rate can induce secondary metabolites synthesis in plants [29]. The reduction in maximum quantum yield (f_v_/f_m_) with increasing production of secondary metabolites as water field capacity was being reduced again demonstrated the possible production of *L. pumila* plant secondary metabolites under increasing plant water stress.

### 2.4. Relative Leaf Water Content

Relative water content was influenced by the interaction effect between variety and ER (*p* ≤ 0.05; Table 3). In both variety var. *alata* and var. *pumila* as field water capacity was reduced from 100% to 25% the relative water content decreased significantly. The reduction in relative water content in var. *pumila* was from 70.83%–94.79% and for var. *alata* was from 72.72%–89.31%. From the correlations in Table 2, it was shown that relative water content had a significant negative correlation with total phenolics (R^2 ^ = −0.854; *p* ≤ 0.05), flavonoids (R^2^ = −0.921; *p* ≤ 0.05) and anthocyanins content (R^2 ^ = −0.865; *p* ≤ 0.05). This data indicate the increased production of plant secondary metabolites under low relative water content in *L. pumila*. A similar obervation to that of the present study was also reported by Schreiner *et al.* [41], whereby they observed enhancement of 2-propenyl- and 3-indolylmethyl glucosinolate concentration in *Brassica carinata* when the relative water content was maintained below 80%. The significant negative correlation of relative water content with plant secondary metabolites was also observed by Xiao *et al.* [42] and Szabo *et al.* [43] in *Populus cathogan* and *Papaver somniferum*, respectively. 

**Table 4:** Interaction effects between varieties and soil water capacity replacement on relative water content and sucrose content.

Varieties	Evapotranspiration	Relative leaf water	Sucrose content
	replacement	content (%)	mg/g dry weight
*Alata*	100%	89.31 ± 1.01 ^b^	27.52 ± 3.01 ^d^
	75%	87.32 ± 0.12 ^b^	32.41 ± 0.93 ^c^
	50%	79.21 ± 0.32 ^c^	64.31 ± 0.83 ^b^
	25%	72.72 ± 0.74 ^d^	63.64 ± 1.12 ^b^
*Pumila*	100%	94.79 ± 0.93 ^a^	27.57 ± 3.15 ^d^
	75%	85.27 ± 0.54 ^b^	35.69 ± 5.16 ^c^
	50%	77.89 ± 1.02 ^c^	67.75 ± 2.11 ^a^
	25%	70.83 ± 2.07 ^d^	66.32 ± 0.07 ^a^

All analyses are mean ± standard error of mean (SEM); *N* = 30 Means not sharing a common letter are significantly different at *p* ≤ 0.05.

### 2.5. Soluble Sugar (Sucrose) Content

The accumulation of soluble carbohydrates in *L. pumila* was influenced by the interaction between variety and ER status and followed a descending order of 50% soil water capacity >25% field capacity >75% field capacity >100% field capacity in both variety var. *alata* and var. *pumila* (Table 4). In the experiments, the soluble sugar contents at var. *alata*-100% ER, var. *alata* −75% ER, var. *alata*-50% ER, var. *alata* −25% ER, var. *pumila*-100% ER, var. *pumila* −75% ER and var. *pumila* −25% ER were 27.57, 32.41, 64.31, 63.64, 27.57, 35.69 and 66.32 mg sucrose/g dry weight, respectively, compared to 67.75 mg sucrose/g dry weight at var. *pumila* −50% ER. The present results suggest that at low field water capacity, especially at 50% ER, the production of soluble sugar was enhanced. Jaafar [44] regarded the accumulation of soluble carbohydrate as due to a reduction in soluble sugar transportation under water stress. Meanwhile, Ghasemzadeh *et al.* [45] described the accumulation of carbohydrates as a signal of an increase in production of secondary metabolites that enhances the medicinal quality of plants. The present finding was in agreement with Guo *et al.* [46] who found an increase in sucrose content corresponded with the enhanced production of ascorbic acid, glucosinolates, sulforaphane, anthocyanins, total phenolics and increased antioxidative activities in broccoli sprouts. The positive correlation between carbohydrate content and antioxidative properties in plants were also reported by other researchers [47,48]. The current results indicate the exposure of *L. pumila* to high water stress can enhance the health promoting effects of this plant with the increase in total phenolics, flavonoids and anthocyanin contents.

### 2.6. Total Chlorophyll Content

**Figure 1:**
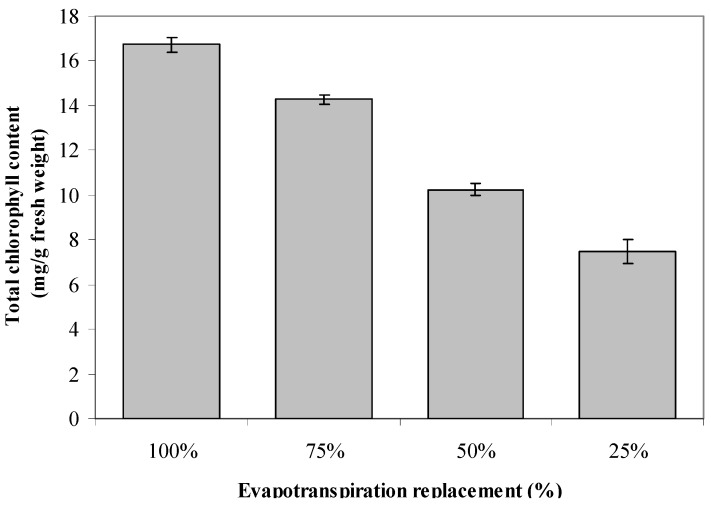
The effects of different evapotranspiration replacement on chlorophyll content of *L. pumila*. N = 60. Bars represent standard error of differences between means (SEM).

The production of total chlorophyll content was dramatically influenced by the SWC (*p* ≤ 0.01; Figure 1). As the levels of water stress became serious (going from 100% to 25%) with replacement of evapotranspiration, the production of total chlorophyll content was found to decrease. The increase in chlorophyll production with increasing water stress has been observed by Jaafar [44] in bell paper plants. It was found from the correlation analysis (Table 2) that total chlorophyll was significantly (*p* ≤ 0.01) and negatively related to the production of secondary metabolites. The negative relationship between chlorophyll content and secondary metabolite production fits well with the protein competition model (PCM) proposed by Jones and Hartley [49], whereby the secondary metabolites content is controlled by the competition between protein and secondary metabolites biosynthesis pathways and the metabolites regulation. The negative relationship between the secondary metabolites and chlorophyll is a sign of gradual switch-off of investment from protein to polyphenols production [50]. The present study indicated that when production of total chlorophyll was down-regulated under water stress condition it may be the signal for increased production of total phenolics, flavonoids and anthocyanins in *L. pumila*.

### 2.7. Phenylalanine Ammonia-Lyase Activity

**Figure 2:**
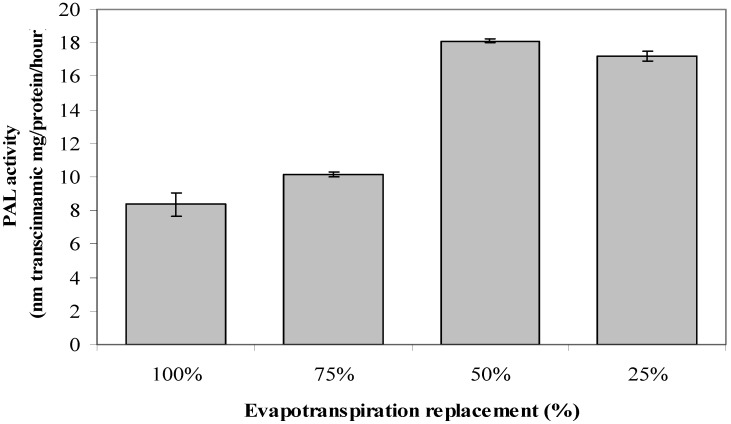
The effects of different evapotranspiration replacement on PAL activity of *L.pumila*. N = 60. Bars represent standard error of differences between means (SEM).

The PAL activity in *L. pumila* was influenced by ER (*p* ≥ 0.05; Figure 2). No varietal effects or any interaction with field capacity was observed. PAL activity was found to be the highest (18.11 nM *trans*-cinnamic mg/protein/h) when at 50% ER, and the lowest activity was demonstrated at 100% ER which registered a value of 8.35 nM *trans*-cinnamic mg/protein/h. The increase in the production of secondary metabolites in the present work could be related to the increase in PAL activities under low water field capacity replacement. Correlation analysis showed that PAL had established a significant (*p* ≤ 0.05) positive relationship with total phenolics (R^2^ = 0.932; *p* ≤ 0.05), flavonoids (R^2^ = 0.876; *p* ≤ 0.05) and anthocyanin (R^2^ = 0.812; *p* ≤ 0.05), suggesting an up-regulation of plant secondary metabolite production with increased PAL activity. This is basically due to the fact that PAL is an enzyme, which synthesizes a precursor for total phenolics and flavonoids biosynthesis. The high water stress in the present study might have increased the availability of the phenylalanine (Phe) pool as less protein was used for plant maintenance under high water stress hence, more Phe is available for the production of secondary metabolites [51,52]. These results suggested that an up-regulation of plant secondary metabolites production in *L. pumila* under high water stress might be due to increased in PAL activity due in turn to increased availability of Phe under stress conditions.

### 2.8. Lipid Peroxidation Activity

**Figure 3:**
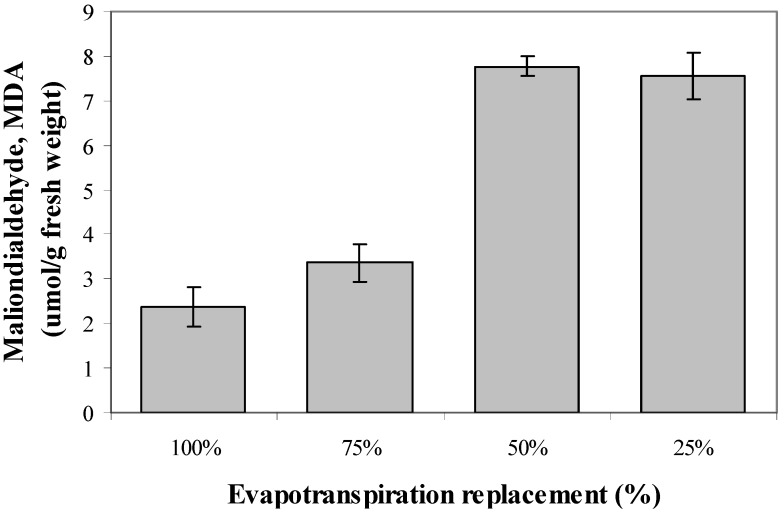
The effects of different evapotranspiration replacement on lipid peroxidation activity of *L.pumila*. N = 60. Bars represent standard error of differences between means (SEM).

Malondialdehyde (MDA) is a highly reactive three carbon dialdehyde produced as a byproduct of polyunsaturated fatty acid peroxidation and arachidonic acid metabolism. The production of MDA was influenced by SWC imposed onto *L. pumila* seedlings (*p* ≤ 0.01; Figure 3). It was observed that as the levels of water stress was enhanced from 100% to 25% water field capacity replacement the production of MDA was also increased. This suggests that as water stress levels increased, the oxidative stress in *L. pumila* cells and tissues were enhanced, thus implying occurrence of lipid peroxidation in *L. pumila* under high water stress. From the correlations in Table 2, the MDA production has established significant positive correlations with total phenolics (R2 = 0.911; *p* ≤ 0.05) and total flavonoids (R^2^ = 0.885; *p* ≤ 0.05), indicating that an increase in MDA might be involved in the up-regulation of the secondary metabolite production under high water stress in *L. pumila*. The formation of MDA was considered as a measure of lipid peroxidation that was induced by a high water stress level [49]. MDA, a decomposition product of polyunsaturated fatty acid hydroperoxides, has been utilized very often as a suitable biomarker for oxidative stress. Usually the increase in lipid peroxidation was simultaneously accompanied by an increase in hydrogen peroxide levels, but in the present study hydrogen peroxide content was not measured. Hydrogen peroxide may function as a signal for the induction of plant defence systems and this could enhance secondary metabolite production [53]. The result suggests that the oxidative stress is a pre-requisite for secondary metabolite synthesis in *L. pumila* under high water stress.

## 3. Experimental

### 3.1. Plant Material and Maintenance

The experiments were carried out in glasshouse at Field 10, University Agriculture Park, Faculty of Agriculture Glasshouse Complex, Universiti Putra Malaysia (longitude 101° 44' N and latitude 2° 58' S, 68 m above sea level) with a mean atmospheric pressure of 1.013 kPa. Three-month old *L. pumila* seedlings were left for a month to acclimatize in a nursery until ready for the treatments. When the seedlings had reached 4 months of age they were fertilized with NPK blue at 15 g per plant. The seedlings were planted in soilless medium containing coco-peat, burnt paddy husk and well composted chicken manure in 5:5:1 (v/v) ratio in 25 cm diameter polyethylene bags. Day and night temperatures in the greenhouse were maintained at 27–30 °C and 18–21 °C, respectively, and relative humidity from 50 to 60%. The experiment was based on a factorial Randomized Complete Block Design (RCBD) with three replicates. The first factor was two varieties of *L. pumila* (var. *alata and* var. *pumila*) and the second factor was water stress treatment based on evapotranspiration replacement (ER). There were four levels of evapotranspiration replacement, *i.e*., (100% ER; well watered), (75% ER; moderate water stress), (50% ER; high water stress treatment) and (25% ER; severe water stress) [54]. All polybags initially receives equal volumes of water to maintain them to near to the predetermined polybag capacity (0.6 litre per 2 L of media) and moisture lost by ER was replaced on alternate days (100%, 75%, 50% and 25%). Each combination treatment consisted of 10 plants totaling a sum of 240 plants used in the experiments. Plants were harvested at 12 weeks after planting [55].

### 3.2. Total Phenolics and Flavonoids Quantification

The method of extraction and quantification for total phenolics and flavonoids contents followed after Ibrahim and Jaafar [56]. An amount of ground tissue samples (0.1 g) was extracted with 80% ethanol (10 mL) on an orbital shaker for 120 min at 50 °C. The mixture was subsequently filtered (Whatman™ No.1), and the filtrate was used for the quantification of total phenolics and total flavonoids. Folin-Ciocalteu reagent (diluted 10-fold) was used to determine the total phenolics content of the leaf samples. Two hundred µL of the sample extract was mixed with Follin–Ciocalteau reagent (1.5 mL) and allowed to stand at 22 °C for 5 min before adding NaNO_3_ solution (1.5 mL, 60 g L^−1^). After two hours at 22 °C, absorbance was measured at 725 nm. The results were expressed as mg g^−1^ gallic acid equivalent (mg GAE g^−1^ dry sample). For total flavonoids determination, a sample (1 mL) was mixed with NaNO_3_ (0.3 mL) in a test tube covered with aluminium foil, and left for 5 min. Then 10% AlCl_3_ (0.3 mL) was added followed by addition of 1 M NaOH (2 mL). Later, the absorbance was measured at 510 nm using a spectrophomtometer with rutin as a standard (results expressed as mg g^−1^ rutin dry sample).

### 3.3. Anthocyanins Content

Anthocyanin content was determined according to Bharti and Khurana [57]. Fresh leaves (1 g) were added in acidic methanol (10 mL, 1% v/v HCl) and incubated overnight. Anthocyanin was partitioned from chlorophyll with chloroform (10 mL), followed by double deionised water (9 mL). The test tubes containing the samples were shaken gently and allowed the mixture to settle. The absorbance was read at 505 nm. Petunidin was used as a standard. Anthocyanin content was recorded as mg/g petunidin fresh weight.

### 3.4. Leaf Gas Exchange Measurement

The measurement was obtained from a LICOR 6400 Portable Photosynthesis System closed infra-red gas analyzer (IRGA, Licor Inc. Nebraska, USA). Prior to use, the instrument was warmed for 30 min and calibrated with the ZERO IRGA mode. Two steps are required in the calibration process: first, the initial zeroing process for the built-in flow meter; and second, zeroing process for the infra-red gas analyzer. The measurements used optimal conditions set of 400 µmol mol^−1^ CO_2_ 30 °C cuvette temperature, 60% relative humidity with air flow rate set at 500 cm^3^ min^−1^, and modified cuvette condition of 800 µmol m^−2^ s^−1^ photosynthetically photon flux density (PPFD). The measurements of gas exchange were carried out between 09:00 to 11:00 a.m. using fully expanded young leaves numbered three and four from plant apex to record net photosynthesis rate (A). The operation was automatic and the data were stored in the LI-6400 console and analyzed by the *Photosyn Assistant* software (Version 3, Lincoln Inc.). Several precautions were taken to avoid errors during measurements. Leaf surfaces were cleaned and dried using tissue paper before enclosed in the leaf cuvette [58]. The light response curve was produced followed procedures from Ibrahim and Jaafar [29] to generate the apparent quantum yield and dark respiration rate.

### 3.5. Chlorophyll Fluorescences Measurement

Measurements of chlorophyll fluorescence were taken from fully expanded leaf of the second leaves. Leaves were darkened for 15 min by attaching light-exclusion clips to the central region of the leaf surface. Chlorophyll fluorescence was measured using a portable chlorophyll fluorescence meter (Handy PEA, Hansatech Instruments Ltd, Kings Lynn, UK). Measurements were recorded up for 5 S [29]. The fluorescence responses were induced by emitting diodes. Measurement of f_O_ (initial fluorescence), f_M_ (maximum fluorescence) and f_V_ (variable fluorescence) were obtained from this procedure. f_V_ is derived as the differences between f_M_ and f_O_.

### 3.6. Relative Leaf Water Content

Relative water content (RWC) was estimated by a modification of the method of Weatherley [59] and calculated as RWC = 100 × [FW − DM] / [TW − DM]. FW and DM denote fresh weight (g) and dry weight (g). Turgid weight (TW) was calculated after fully hydrating fresh leaves in darkness at 4 °C for 24 h. Results were expressed as percentages.

### 3.7. Sucrose Determination

Sucrose was measured spectrophotometrically using the method of Ibrahim and Jaafar [60]. Samples (0.5 g; 0.25 mm) were placed in 15 mL conical tubes, and distilled water added to make up the volume to 10 mL. The mixture was then vortexed and later incubated for 10 min. Anthrone reagent was prepared using anthrone (0.1 g) that was dissolved in 95% sulphuric acid (Fisher Scientific, USA, 50 mL). Sucrose was used as a standard stock solution to prepare a standard curve for the quantification of sucrose in the sample. The mixed sample of ground dry sample and distilled water was centrifuged at a speed of 3,400 rpm for 10 min and then filtered to get the supernatant. A sample (4 mL) was mixed with anthrone reagent (8 mL) and then placed in a water-bath set at 100 °C for 5 min before the sample was measured at an absorbance of 620 nm using a spectrophotometer model UV160U (Shimadzu Scientific, Kyoto, Japan). The soluble sugar in the sample was expressed as mg sucrose g^−1^ dry sample.

### 3.8. Chlorophyll Content

Total chlorophyll content was measured by method from Jaafar *et al*. [61] using fresh weight basis. Prior to each destructive harvest each seedling was analyzed for the leaf chlorophyll relative reading (SPAD meter 502, Minolta Inc., Alameda, CA, USA). The leaves of *L. pumila* with different greenness (yellow, light green and dark green) were selected for analysis and total leaf chlorophyll content was analyzed. For each type of leaf greenness, the relative SPAD value was recorded (five points/leaf) and the same leaves sampled for chlorophyll content determination. Leaf disk 3 mm in diameter was obtained from leaf sample using a hole puncher. For each seedling the measurement was conducted on the youngest fully expanded leaves on each plant, generally the second or third leaf from the tip of the stem was used. The leaf disks were immediately immersed in acetone (20 mL) in an aluminum foil-covered glass bottle for approximately 24 h at 0 °C until all the green colour had bleached out. Finally, the solution (3.5 mL) was transferred to measure absorbances at 664 and 647 nm using a spectrometer (UV-3101P, Labomed Inc., Palmer, Alaska, AK, USA). After that the least squares regression was used to develop predictive relation between SPAD meter readings and pigment concentrations (mg g^−1^ fresh weight) obtained from the chlorophyll destructive analysis.

### 3.9. Phenylalanine-Ammonia-Lyase (PAL) Activity Determination

Phenylalanine-ammonia-lyase (PAL) activity was measured using the method described by Martinez and Lafuente [62]. The enzyme activity was determined by measuring spectrophotometrically the production of *trans*-cinnamic acid from L-phenylalanine. Enzyme extract (10 µL) was incubated at 40 °C with 12.1 mM L-phenylalanine (90 µL, Sigma) that were prepared in 50 mM Tris-HCl, (pH 8.5). After 15 min of reaction, *trans*-cinnamic acid yield was estimated by measuring increase in the absorbance at 290 nm. Standard curve was prepared by using a *trans*-cinnamic acid standard (Sigma) and the PAL activity was expressed as nM *trans*-cinnamic acid µg^−1^ protein h^−1^.

### 3.10. Malondialdehyde (MDA) Content Determination

Lipid peroxidation of plant parts was estimated by the level of malondialdehyde (MDA) production using the thiobarbituric acid (TBA) method as described by Ibrahim and Jaafar [63]. One gram of ground (0.25 mm) plant sample was homogenized with a mortar and pestle in 0.5% trichloracetic acid (TCA, 1 mL). The homogenate was centrifuged at 9,000 rpm for 20 min. The supernatant (0.5 mL) was mixed with 20% TCA (2.5 mL) containing 0.5% TBA and heated in a boiling water bath for 30 min and allowed to cool in an ice bath quickly. The supernatant was centrifuged at 9,000 rpm for 10 min, and resulting supernatant was used for determination of MDA content. Absorbance at 532 nm was recorded.

### 3.11. Statistical Analysis

Data were analyzed using analysis of variance by SAS version 17. Mean separation test between treatments was performed using Duncan multiple range test and standard error of differences between means was calculated with the assumption that data were normally distributed and equally replicated [64,65,66].

## 4. Conclusions

Our results indicate that the manipulation of soil water capacity may be an effective method to increase the expression of secondary metabolites compounds in *L. pumila*. Higher total flavonoids, phenolics, and anthocyanin levels were demonstrated in *L. pumila* when soil water capacity was at 50% (high water stress). The significant negative correlations of production of total flavonoids, phenolics and anthocyanins content with photosynthesis, apparent quantum yield and maximum efficiency of photosystem II (f_v_/f_m_) indicate the occurrence of the up-regulation of production of CBSM under reduced photosynthetic capacity under high water stress. The increase in the production of *L. pumila* secondary metabolites under low water field capacity might be due to enhancement of PAL and MDA activity that were shown to have significant positive correlations with plant secondary metabolites. Under high and severe water stress it was also noted that the production of chlorophyll was reduced significantly, indicating the increased production of plant secondary metabolites. 
